# Long-term exposure to carcinoma-associated fibroblasts makes breast cancer cells addictive to integrin β1

**DOI:** 10.18632/oncotarget.25183

**Published:** 2018-04-24

**Authors:** Angela Dittmer, Jürgen Dittmer

**Affiliations:** ^1^ Clinic for Gynecology, Martin Luther University Halle-Wittenberg, Halle/Saale, Germany

**Keywords:** carcinoma-associated fibroblast, anti-estrogen resistance, integrin β1, tumor heterogeneity, breast cancer

## Abstract

We studied the long-term effect of stromal factors on the development of fulvestrant-resistance (FR) and fulvestrant-induced dormancy (D). Sublines established from stroma-treated FR-cells (C-FR cells) and D-cells (C-D cells) show permanently high expression of integrin β1 as well as Bcl-3 and P-STAT3 (C-FR) or IGF1R (C-D). Yet, cells fail to withstand fulvestrant better and do not migrate or grow faster than control cells. Instead, C-D cells rely on stromal factors to perform as well as control cells. In addition, C-FR cells adapted to integrin β1 for growth in 3D cultures. These data suggest that long-term exposure to stromal factors leads to addiction rather than better performance in cellular activities. We also found that morphologically distinct breast cancer cell line subpopulations share key responses to stromal factors suggesting that intratumoral heterogeneity may play a minor role in the interaction between breast cancer and stromal cells.

## INTRODUCTION

Tumor-residing stroma cells play an important role in tumor progression [1]. In particular, carcinoma-associated fibroblasts (CAFs) have been demonstrated to affect tumor progression in many ways [2].This includes protection against cancer-targeted drugs [3]. We have previously shown that CAFs and bone marrow-derived mesenchymal stem cells (MSCs), which can differentiate to CAFs, have similar protecting effects on ERα-positive breast cancer cells when they were treated with the anti-estrogen fulvestrant (ICI 182,780) [4]. These effects were found to be mediated by factors secreted by the stromal cells. A cocktail of CAF/MSC-secreted factors induced the expression of a number of cancer-related proteins, such as integrin β1, P-AKT, P-STAT3 (signal transducer and activator of transcription 3), CAIX (carbon anhydrase IX), IGF1R (insulin-like growth factor 1 receptor) and Bcl-3 (B-cell leukemia/lymphoma 3), in breast cancer cells. Of these factors, Bcl-3 could be linked to fulvestrant resistance. IGF1R and the PI3K (phosphoinositol-3-kinase)/AKT pathway have been previously associated with resistance to the anti-estrogen tamoxifen [5]. CAFs are able to induce tamoxifen resistance by secreting fibronectin, which stimulates its ligand integrin β1 to activate signaling pathways, such as the PI3K/AKT pathway [6].

Studies linking stroma cells to drug resistance are mostly conducted with a short-term co-culture approach. However, *in vivo*, it is likely that the majority of tumor cells are permanently exposed to stromal cells leading to long-term effects that may be missed by short-term approaches. E.g., long-term exposure to stromal cells may initiate a selection process within the tumor cell population leading to the expansion of certain clones. This was shown for triple-negative breast cancer that responds to IGF/CXCL12-secreting CAFs by increasing the proportion of Src kinase-hyperactive cancer cells with disseminating potential [7]. There is also evidence that environmental factors influence the epigenome by silencing or overactivating genes [8].

Based on these finding we sought to analyze the long-term effect of conditioned medium (CM) from CAFs on breast cancer cells in the presence of fulvestrant. Long-term exposure of ERα-positive breast cancer cells to fulvestrant, which not only blocks ERα activity, but also downregulates its expression [9], leads to massive cell death. Surviving cells may be either proliferative fulvestrant-resistant cells or cells in a dormant, mitogenically inactive state. Dormancy plays an important role in metastasis [10, 11]. It is triggered by a dormancy-permissive microenvironment and protects cells against cancer-targeting drugs.

Intratumoral heterogeneity in breast cancer is gaining increasing attention [12, 13]. Intratumoral heterogeneity is caused by the evolution of subpopulations with distinct phenotypes. Different subpopulations have also been identified in breast cancer cell lines [14, 15]. Therefore, it was also of interest to study whether subpopulations of a cell line would react differently to stromal cells.

We studied the impact of long-term and short-term exposure to CAF-CM on fulvestrant sensitivity on MCF-7 cells by using fulvestrant-resistant sublines and sublines generated from cells that tolerated fulvestrant in a quiescent state. We found tremendous differences in the effects of long-term and short-term treatment with CAF-CM and present data suggesting that long-term treatment leads to addiction to integrin β1.

## RESULTS

### Isolation and characterization of two morphologically distinct cell types within the MCF-7 cell line

To address the importance of intra-tumoral heterogeneity for the responses of breast cancer cells to CAFs, we first isolated and characterized subpopulations of MCF-7 cells. When seeded MCF-7 cells at low density, two distinct phenotypes of cells were found to form colonies. From each phenotype a subline was established, which we called AnD3 and AnD5 (Supplementary Figure 1). Confirmation of the MCF-7 genetic background of both sublines was done by SNP-analysis-based authentication and by verification of the existence of the *pik3ca*-G1633A mutation (data not shown) inherent to MCF-7 cells [16].

**Figure 1:**
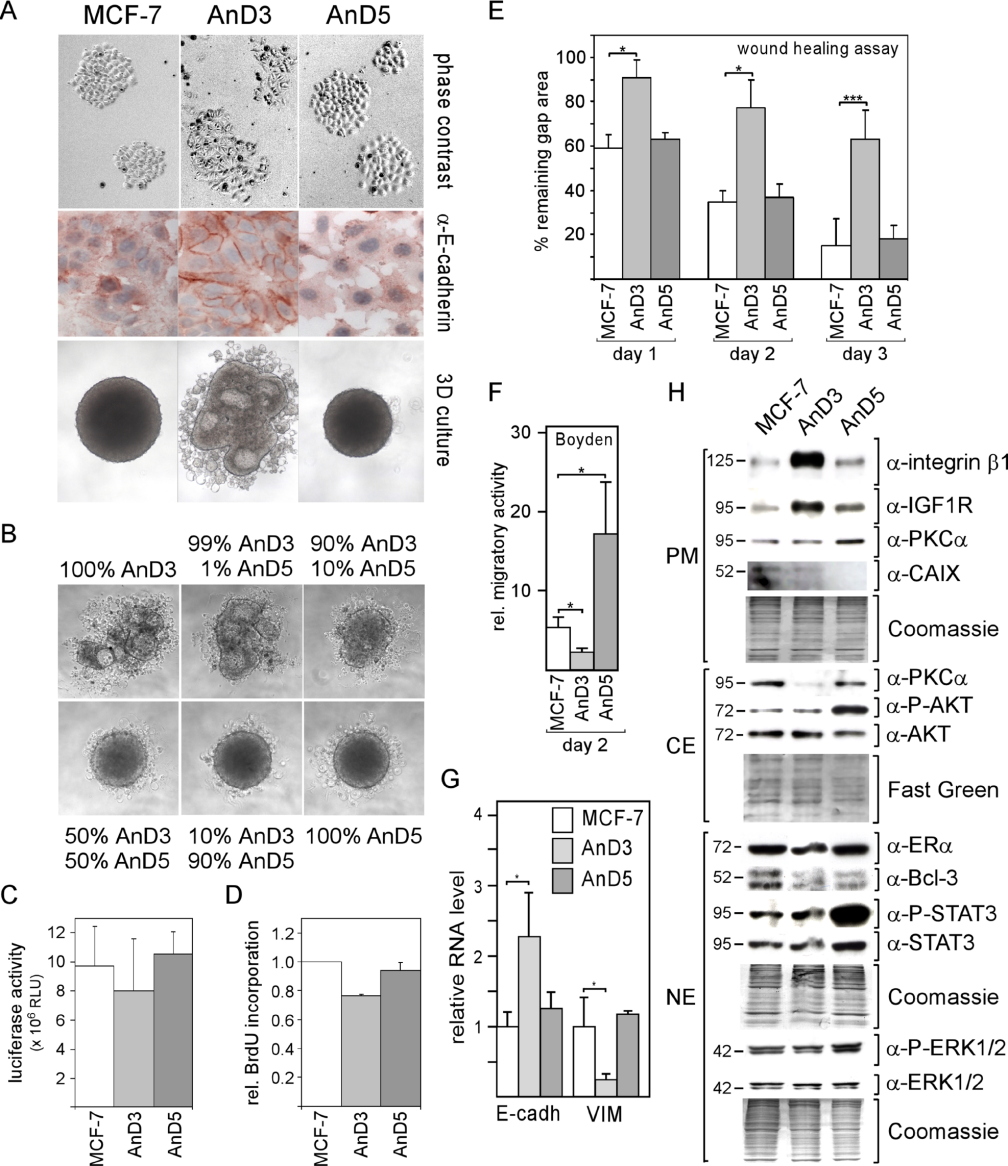
MCF-7 cell line-derived sublines AnD3 and AnD5 show strong difference in cellular morphology, spheroid formation ability and migration activity (**A**) Phase contrast images of individual colonies of adherent MCF-7, AnD3 and AnD5 cells (top), immunocytochemical analysis for E-cadherin expression (center) and cell aggregation after four days in 3D suspension cultures (bottom). (**B**) AnD5 cells dominate cell aggregation pattern in 3D cultures when mixed with AnD3 cells. AnD3 and AnD5 cells were mixed at ratios as indicated and kept in 3D cultures for four days. (**C**) Cells were grown for six days in 2D adherent cultures, before cell mass was determined by a luciferase-based growth assay. (**D**) Bromo-deoxyuridine (BrdU)-based proliferation assay. Cells were grown for three days, before DNA was analyzed for BrdU incorporation. (**E**) Wound healing assay. Gap closure was measured on day 1, 2 and 3 after the wound had been set. (**F**) Boyden chamber assay. The number of cells that migrated through the membrane was measured two days after the experiment had been started. (**G**) RNA expression of E-cadherin and vimentin as measured by Q-RT-PCR. (**H**) Protein expression of selected proteins as determined by Western blot analysis. Protein loading was checked by Commassie Blue or Fast Green. PM, CE and NE denote plasma membrane, cytosolic and nuclear extract, respectively.

AnD3 cells show an epithelial-like appearance and high E-cadherin protein abundance at the cell-cell boundaries (Figure 1A). In contrast, like the majority of MCF-7 cells, AnD5 cells display a round-shaped morphology and a more diffuse intracellular E-cadherin distribution. AnD3 cells also differ from AnD5 and MCF-7 cells in their aggregation behavior in 3D suspension cultures. AnD3 cells form many small, differently shaped aggregates, which often show luminal spaces, rather than well-shaped spheroids as found with AnD5 and MCF-7 cells, whereby AnD5 spheroids are smaller than MCF-7 spheroids. In the presence of only 10% AnD5 cells, AnD3 cells tend to form spheroid-like structures (Figure 1B) suggesting that AnD5 cells dominate aggregation behavior in 3D cultures. If the AnD5 fraction was 50% or higher, well-shaped spheroids were formed. In terms of growth and proliferation, AnD3 cells are somewhat less active than AnD5 and MCF-7 cells (Figure 1C, 1D). In wound healing and Boyden chamber assays, AnD3 cells show a much lower migration activity than AnD5 and MCF-7 cells (Figure 1E, 1F). AnD5 and MCF-7 cells are equally fast in closing the wound, whereas, in Boyden chamber assay, AnD5 cells migrate faster than MCF-7 cells. The different migration pattern of the three cell lines coincided with a more than 2-fold higher E-cadherin RNA level and a 4-fold lower RNA expression of vimentin in AnD3 cells as compared with AnD5 and MCF-7 cells (Figure 1G). In terms of expression of selected proteins, AnD3 cells produce more integrin β1 and IGF1R than AnD5 and MCF-7 cells, whereas AnD5 cells show a higher phosphorylation status of STAT3, AKT and ERK1/2 than AnD3 and MCF-7 cells (Figure 1H). Collectively, these data show that AnD3 cells are epithelial-like tumor cells with high E-cadherin expression and low migratory activity, whereas AnD5 cells represent a highly motile, more proliferative fraction of the MCF-7 cell line and accordingly show high activities in key signaling pathways linked to cell growth and migration. These data also show that AnD5 cells share most of the features of MCF-7 cells suggesting that they comprise the majority of the MCF-7 population.

### AnD3 and AnD5 cells share key responses to CAF-CM

**Figure 2:**
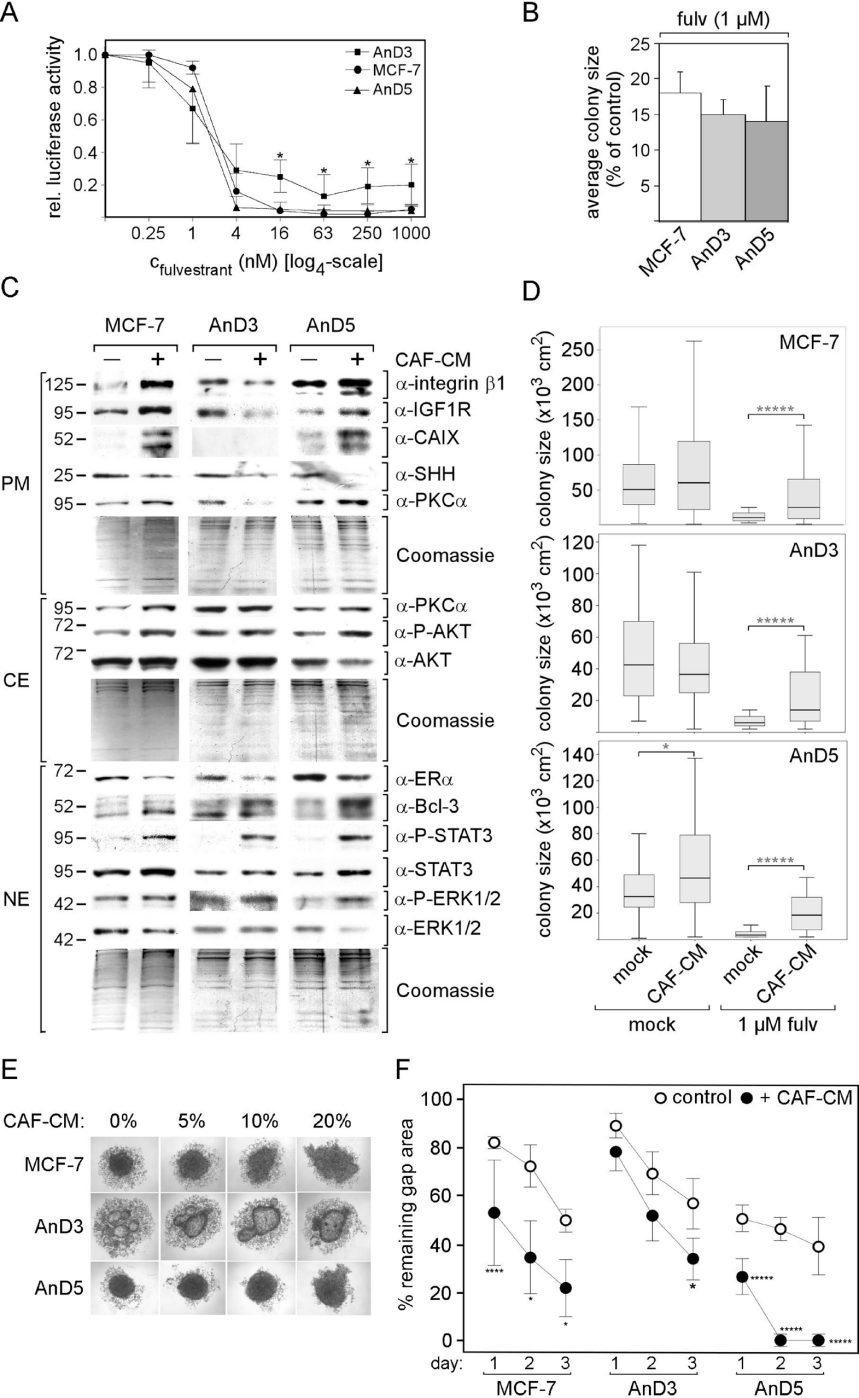
Responses of AnD3 and AnD5 cells to fulvestrant and CAF-CM (**A**) Growth activities of AnD3, AnD5 and MCF-7 cells in the presence of different concentrations of fulvestrant or in the absence of fulvestrant. Cell mass was determined after six days of growth by a luciferase-based assay from at least three independent experiments. For statistical analyses by student *t*-test, growth of AnD3 or AnD5 cells was compared with growth of MCF-7 parental cells for each condition. (**B**) Colony growth assay. Growth assay was done at low density which forces cells to form individual colonies. After six days of growth, colonies sizes were measured and, for each cell line, the relative average colony size (in the presence vs. absence of fulvestrant) was determined from three independent experiments. (**C**) Effect of 3-day-exposure of MCF-7, AnD3 and AnD5 cells to CAF-CM (20%) on the expression of selected proteins as determined by Western blot analysis. Protein loading was checked by Commassie Blue. PM, CE and NE denote plasma membrane, cytosolic and nuclear extract, respectively. (**D**) Effect of 6-day-exposure of CAF-CM (20%) on colony size of MCF-7, AnD3 and AnD5 cells in the presence or absence of 1µM fulvestrant. For each condition, the sizes of 50 colonies were measured. Statistical analyses were performed by Mann-Whitney-U-test. (**E**) Effect of increasing concentrations of CAF-CM (given as percentage added to the medium) on cell aggregation after three days of incubation in 3D suspension culture. (**F**) Wound healing assay in the presence and absence of 20% CAF-CM in the medium. Gap closure was measured on day 1, 2 and 3 after the wound had been set.

We next analyzed the effect of fulvestrant on growth of AnD3 and AnD5 cells. Exposing cells to different concentrations of fulvestrant for six days revealed that 4 nM fulvestrant was sufficient to kill almost all AnD5 cells, whereas 16 nM fulvestrant was required to eliminate MCF-7 cells (Figure 2A). In contrast, 15–20% of AnD3 cells survived even higher concentrations of fulvestrant (≥ 4 nM). However, when seeded at much lower density which leads to the formation of many individual colonies, all cell lines showed similar sensitivity to 1 µM fulvestrant (Figure 2B). After six days of incubation, average colony size was reduced by ∼85% in the presence of fulvestrant compared to the control condition.

Responses of the two sublines to short-term CAF-CM treatment were analyzed by protein expression, growth, spheroid formation and migration assays. Focussing on proteins that we have previously found to be reactive to CAF-CM [4], on PKCα (protein kinase Cα), which plays a role in drug resistance in MCF-7 cells [17] and on SHH (sonic hedgehog), a fulvestrant-responsive protein [18] involved in estrogen-dependent regulation of proliferation [19, 20], we found similar as well as different responses by AnD3 and AnD5 cells on protein expression. Like MCF-7 parental cells, AnD3 and AnD5 cells increased their levels of Bcl-3 and P-STAT3 and decreased their expression of ERα and SHH upon short-term exposure to CAF-CM (Figure 2C). On the other hand, while, in AnD5 and MCF-7 cells, CAF-CM upregulated the levels of integrin β1, IGF1R, CAIX, P-AKT and plasma membrane-bound PKCα, such changes were not found with AnD3 cells and CAF-CM rather decreased the expression of integrin β1, IGF1R and PKCα in these cells. In growth assays, both sublines behaved similar to the MCF-7 parental cell line and grew much better in fulvestrant-containing medium in the presence of CAF-CM, while gaining little or no benefit from CAF-CM in the absence of fulvestrant (Figure 2D). This finding fits well to the increased level of Bcl-3, the protein responsible for CAF-CM-induced protection against fulvestrant [4]. In the spheroid formation assay, all three cell lines showed changes in cell aggregation pattern in response to CAF-CM (Figure 2E). These changes are likely caused by CAF-CM-upregulated STAT3 phophorylation (Supplementary Figure 2A, 2B). In wound healing assays, short-term CAF-CM treatment strongly increased migration of AnD5 and MCF-7 cells and to a lesser extent also that of AnD3 cells (Figure 2F).

Collectively, these data suggest that AnD3 and AnD5 cells share key reactions to short-term exposure to CAF-CM, namely increased growth in the presence of fulvestrant, increased migration, altered cell aggregation in 3D cultures and altered expression of proteins linked to these cellular activities, but also show differences in the expression of other proteins. Furthermore, given the high degree of similarities between AnD5 and MCF-7 cells in their responses to CAF-CM, the data support the notion that the AnD5 cell is the major cell type in the MCF-7 cell population.

### Long-term exposure of AnD3 and AnD5 cells to CAF-CM leads to addiction to CAF-CM and permanently increased expression of integrin β1 and IGF1R

**Figure 3:**
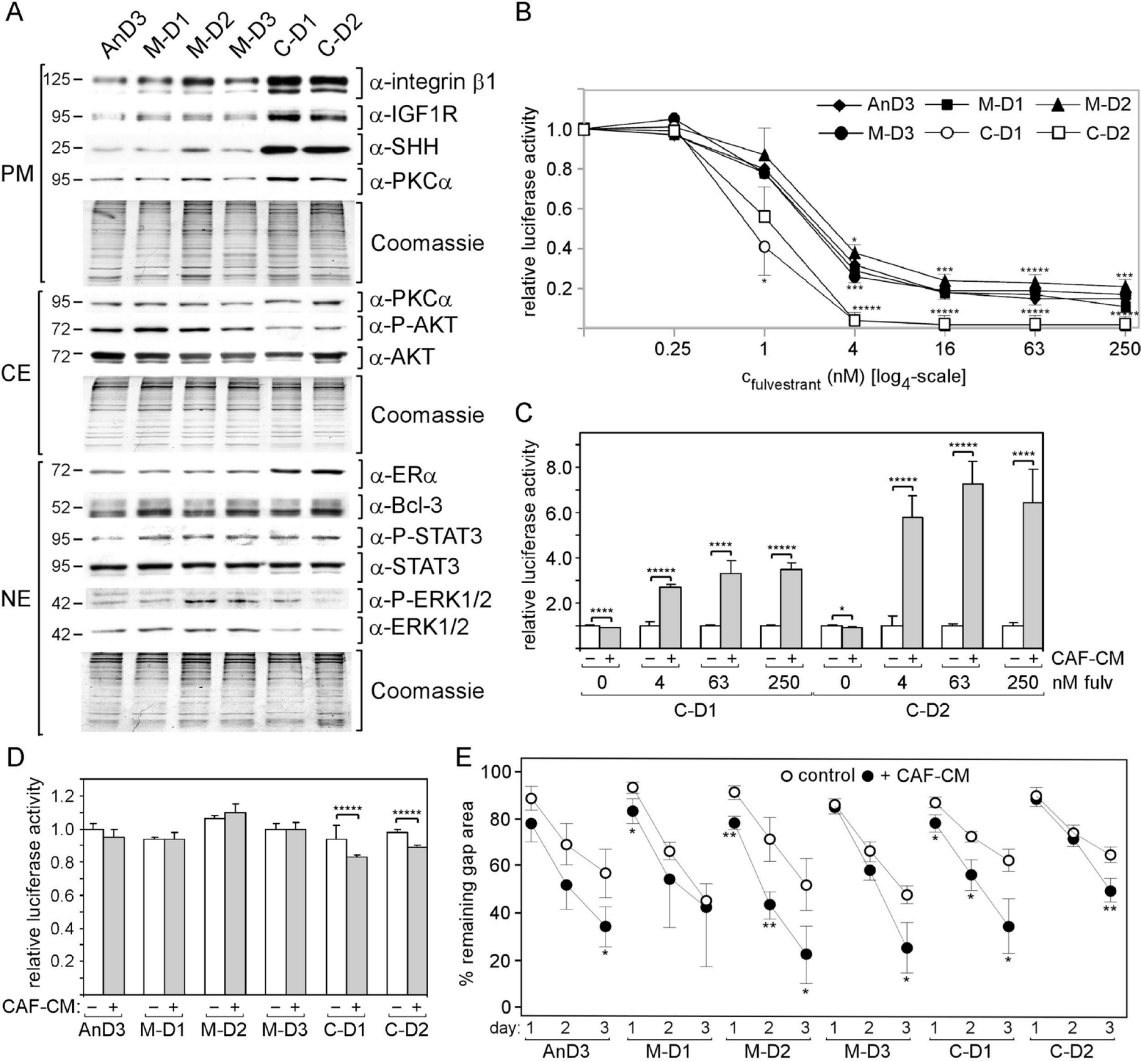
Exposure to CAF-CM during fulvestrant-forced dormancy changes protein expression and fulvestrant sensitivity of AnD3 cells (**A**) Expression of selected proteins in the AnD3 parental line and the AnD3 sublines established from dormant cells in the presence of CAF-CM (C-D1 and C-D2) or MCF-CM (M-D1, M-D2 and M-D3) by Western blot analysis. For comparibility reasons, all cell lines were kept free of CM for five days prior to protein extractions. Protein loading was checked by Commassie Blue. PM, CE and NE denote plasma membrane, cytosolic and nuclear extract, respectively. (**B**–**D**) Growth activities of AnD3 and/or AnD3 subline cells in the presence of different concentrations of fulvestrant or in the absence of fulvestrant. CAF-CM (20%) was added where indicated. Cell mass was determined after six days of growth by a luciferase-based assay. For statistical analyses by student *t*-test, growth activity of each subline was compared to growth activity of the AnD3 parental cell line for each condition (B) or growth activity in the presence vs. absence of CAF-CM was compared (C, D). (**E**) Wound healing assay in the presence and absence of 20% CAF-CM in the medium. Gap closure was measured on day 1, 2 and 3 after the wound had been set.

AnD3 and AnD5 cells did not give rise to proliferating colonies in the presence of 1 µM fulvestrant suggesting that they are unable to generate fulvestrant-resistant cells. However, colonies could be obtained after fulvestrant withdrawal suggesting that some AnD3 and AnD5 cells survived fulvestrant likely by entering dormancy and could still mitotically be re-activated. Sublines established from these colonies were called M-D or C-D sublines, depending on whether they were established in the presence of MCF-CM or CAF-CM (Supplementary Figure 1). Protein expression analyses of AnD3/M-D and AnD3/C-D sublines, which for comparability reasons were performed from cells in the absence of CM, revealed that both C-D cell lines show higher expression of integrin β1, IGF1R, SHH and ERα and lower levels of P-AKT and ERK1/2 as compared to the three M-D cell lines (Figure 3A). Studying fulvestrant sensitivity in the absence of CM revealed a similar dose-response pattern of AnD3/M-D and AnD3 cells, whereby, at higher concentrations of fulvestrant, M-D2 cells was somewhat less sensitive than AnD3 cells (Figure 3B). Compared to AnD3/M-D and AnD3 cells, C-D1 and C-D2 cells were much more sensitive, particularly at higher concentrations of fulvestrant. However, re-addition of CAF-CM strongly improved fulvestrant tolerance and allowed growth activities close to those of AnD3 cells under the same conditions (Figure 3C). In the absence of fulvestrant, all sublines showed similar growth activities (Figure 3D). Also, migration activities of the sublines were similar and were all increased by short-term treatment with CAF-CM (Figure 3E).

**Figure 4:**
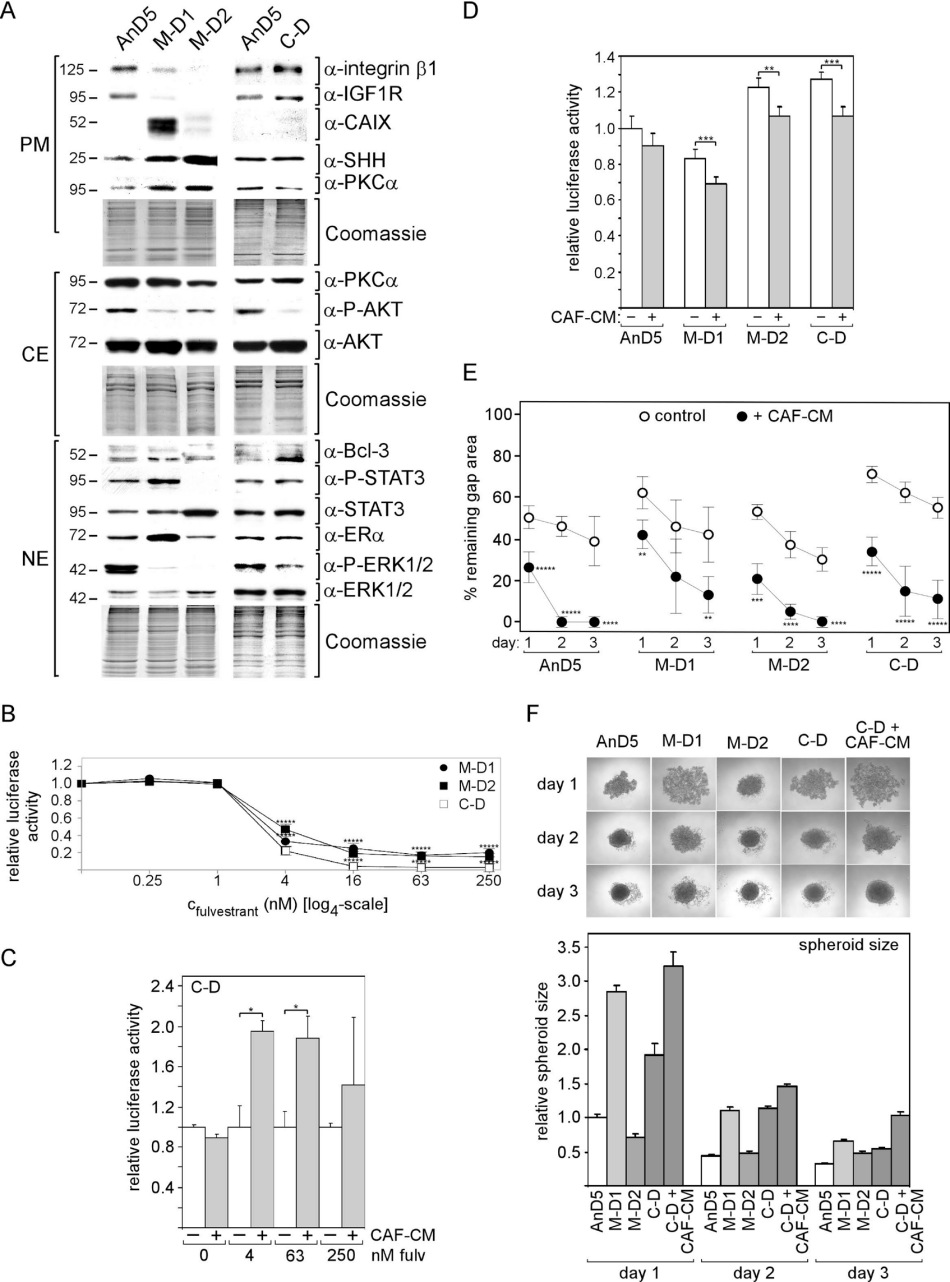
Exposure to CAF-CM during fulvestrant-forced dormancy changes protein expression and fulvestrant sensitivity of AnD5 cells (**A**) Expression of selected proteins in the AnD5 parental line and the AnD5 sublines established from dormant cells in the presence of CAF-CM (**C**–**D**) or MCF-CM (M-D1 and M-D2) by Western blot analysis. For comparibility reasons, all cell lines were kept free of CM for five days prior to protein extractions. Protein loading was checked by Commassie Blue. PM, CE and NE denote plasma membrane, cytosolic and nuclear extract, respectively. (**B**–**D**) Growth activities of AnD5 and/or AnD5 subline cells in the presence of different concentrations of fulvestrant or in the absence of fulvestrant. CAF-CM (20%) was added where indicated. Cell mass was determined after six days of growth by a luciferase-based assay. For statistical analyses by student *t*-test, growth of M-D1 or M-D2 cells was compared to growth of C-D cells for each condition (B) or growth in the presence vs. absence of CAF-CM was compared (C, D). (**E**) Wound healing assay in the presence and absence of 20% CAF-CM in the medium. Gap closure was measured on day 1, 2 and 3 after the wound had been set. Statistical analyses were done by student *t*-test. (**F**) Cell aggregation in 3D cultures in the absence or presence of CAF-CM (20%). Phase contrast images (top) and relative size (bottom) of cell aggregates after 1, 2 and 3 days of incubation.

Two M-D and one C-D subline could be established from AnD5 cells. Like the AnD3/C-D sublines, the AnD5/C-D subline shows higher expression of integrin β1 and IGF1R and lower levels of P-AKT than the corresponding M-D sublines in the absence of CM (Figure 4A). However, unlike the AnD3/C-D sublines, the AnD5/C-D subline express more Bcl-3 and P-ERK1/2 and less SHH than their M-D counterparts. Like AnD3/C-D cells, the AnD5/C-D cells show significantly higher sensitivity to fulvestrant than the M-D cells, when the analysis was done in the absence of CM (Figure 4B). Again, re-addition of CAF-CM was able to increase growth in the presence of fulvestrant, but only by ∼2-fold at 4 and 63 nM fulvestrant (Figure 4C). Growth in the absence of fulvestrant is highest with AnD5/C-D and AnD5/M-D1 followed by AnD5 and AnD5/M-D1 cells (Figure 4D). In all, cases CAF-CM slightly decreases growth in the absence of fulvestrant. In terms of migration, the AnD5/C-D cells are the slowest of all AnD5 subline cells, if CAF-CM is absent. However, in the presence of CAF-CM, they are as fast as M-D1 cells, but still slower than AnD5 parental or M-D2 cells (Figure 4E). Spheroids formation was fastest with AnD5/M-D2 cells (Figure 4F). This result matches well with the observation that these cells lack P-STAT3 (Figure 4A), which we showed to delay spheroid formation (Supplementary Figure 2A, 2B). C-D and M-D1 cells are similar slow in spheroid formation. They either express higher levels of integrin β1 (C-D) or P-STAT3 (M-D1) than AnD5 parental cells.

In conclusion, long-term exposure of dormant AnD3 and AnD5 cells to CAF-CM permanently increased expression of integrin β1 and IGF1R without resulting in a better protection against fulvestrant and higher migratory activity. In the contrary, in the absence of CM, fulvestrant sensitivities of all C-D sublines were substantially higher and migration activity of AnD5/C-D was much lower than those of corresponding control-CM treated cells. Only re-addition of CAF-CM allowed performances in these cellular activities that were close to those of control cells. This suggest that C-D cells got addicted to CAF-CM without gaining an advantage in fulvestrant tolerance and migration activity.

### Development of fulvestrant resistance in the presence of CAF-CM leads to permanently increased levels of integrin β1, P-STAT3 and Bcl-3

We next sought to analyze the effect of CAF-CM on the generation of fulvestrant-resistant (FR) breast cancer cells. Since AnD5 and AnD3 cells did not give rise to fulvestrant-resistant colonies, we used the parental MCF-7 cell line for this study. C-FR sublines were established in the presence of CAF-CM, M-FR sublines in the presence of MCF-CM (Supplementary Figure 1).

**Figure 5:**
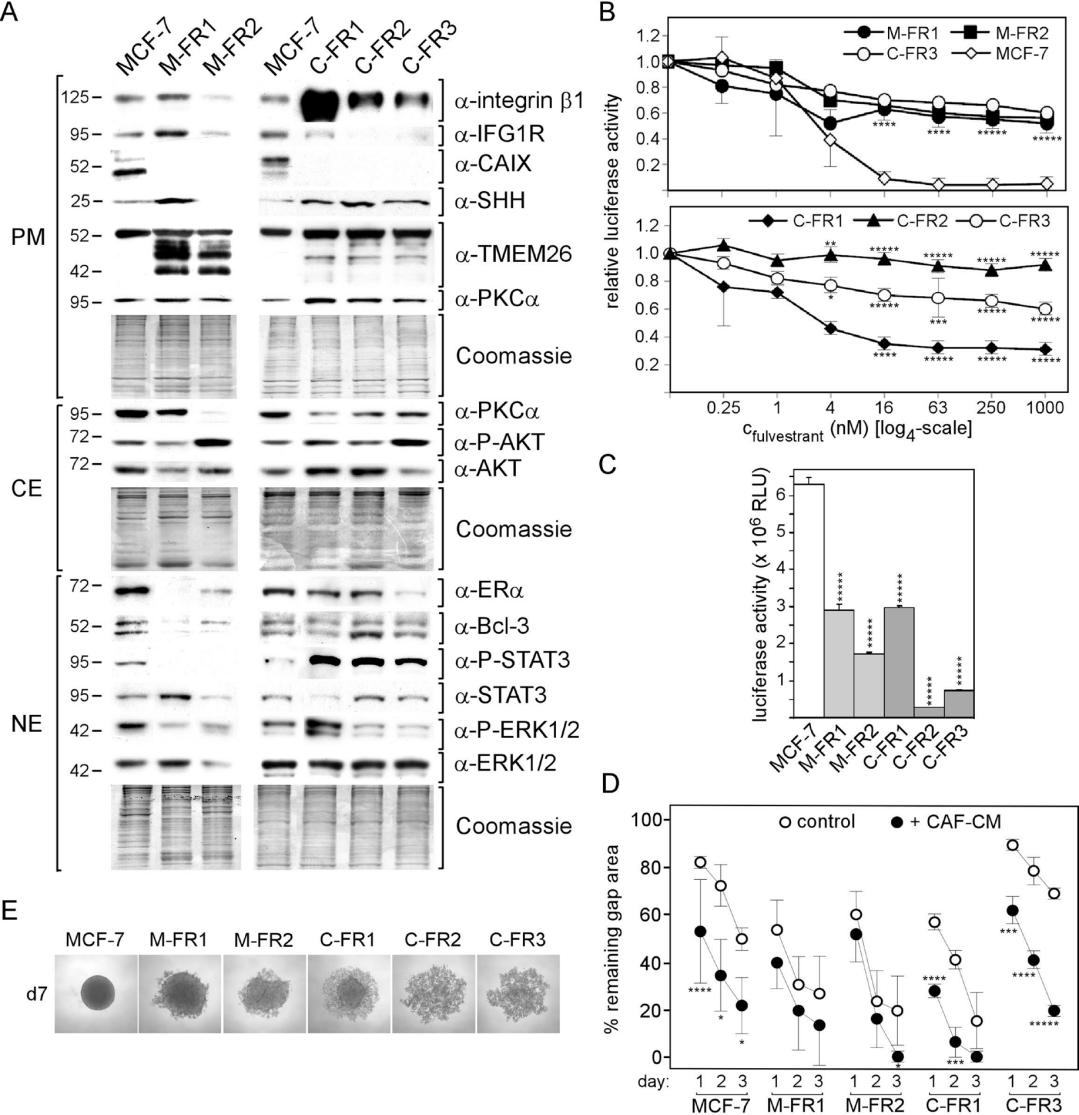
Exposure to CAF-CM during development of fulvestrant resistance leads to permanently high expression of integrin β1 and P-STAT3 levels (**A**) Expression of selected proteins in the MCF-7 parental line and fulvestrant-resistant MCF-7 sublines established in the presence of CAF-CM (C-FR1, C-FR2 and C-FR3) or MCF-CM (M-FR1 and M-FR2) by Western blot analysis. For comparibility reasons, all cell lines were kept free of CM and fulvestrant for five days prior to protein extractions. Protein loading was checked by Commassie Blue. PM, CE and NE denote plasma membrane, cytosolic and nuclear extract, respectively. (**B**, **C**) Growth activities of MCF-7 parental cell lines and its fulvestrant-resistant sublines in the presence of different concentrations of fulvestrant or in the absence of fulvestrant. Cell mass was determined after six days of growth by a luciferase-based assay. For statistical analyses by student *t*-test, growth of all sublines were compared with growth of MCF-7 parental cell line for all conditions. (B) For illustrative reasons, the dose-response curve for the C-FR3 subline is shown in both the upper and lower panel of the figure. (**D**) Wound healing assay in the presence and absence of CAF-CM (20%). Gap closure was measured on day 1, 2 and 3 after the wound had been set. (**E**) Images of the aggregates formed by MCF-7 parental cells and it fulvestrant-resistant derivatives at day 7 in 3D cultures.

Protein analyses of these sublines, all done in the absence of CM and fulvestrant, revealed that all sublines express underglycosylated TMEM26 (transmembrane protein 26), which we have previously found to be linked to fulvestrant resistance [18] (Figure 5A). In addition, all sublines are deficient in the expression of CAIX. Besides these similarities, there are also striking differences between the M-FR and C-FR sublines. Most prominently, all C-FR sublines express much higher levels of integrin β1, P-STAT3 and Bcl-3 than the M-FR sublines.

All FR sublines are much more resistant to fulvestrant than MCF-7 parental cells at concentrations ≥ 16 nM, at which almost all MCF-7 cells die (Figure 5B). Of the FR sublines, C-FR2 is the most resistant FR subline, whereas C-FR1 is the most sensitive FR subline (Figure 5B, lower panel). C-FR3, M-FR2 and M-FR1 show similar dose-response patterns (Figure 5B, upper panel). These data suggest that the presence of CAF-CM during the generation of fulvestrant resistance does not result in higher fulvestrant resistance in general. Also, in terms of growth in the absence of fulvestrant, C-FR sublines are not superior over M-FR sublines (Figure 5C). Rather, two of the C-FR sublines, C-FR2 and C-FR3, show even the slowest growth of all five FR sublines. Among the C-FR sublines, fulvestrant resistance (C-FR2 > C-FR3 > C-FR1) seems to inversely correlate with growth in the absence of fulvestrant (C-FR2 < C-FR3 < C-FR1), which makes sense given that fulvestrant primarily attacks proliferating cells. Taken growth activity into account, only C-FR1 and M-FR1 cells can be directly compared in terms of fulvestrant resistance showing that C-FR1 cells are less resistant than M-FR1 cells. This again suggests that long-term exposure to CAF-CM does not provide better protection against fulvestrant.

We next analyzed migration activities and spheroid formation abilities of the different FR sublines. In wound healing assays, which we run with MCF-7 cells and with all FR sublines except for C-FR2 cells, as the latter do not form proper monolayers, C-FR1, M-FR1 and M-FR2 cells migrated faster than MCF-7 cells (Figure 5D). C-FR3 cells migrated the slowest. Interestingly, exposure to CAF-CM substantially increased migration of C-FR1, C-FR2 and MCF-7 cells, but had only little effect on the M-FR1 and M-FR2 cells. In the presence of CAF-CM, C-FR1 cells, which express the highest level of integrin β1 (Figure 5A), are the fastest of all tested FR cells (Figure 5D). In 3D suspension cultures, all FR sublines showed defects in the ability to form proper spheroids (Figure 5E). Seven days of incubation were needed for the two M-FR sublines to aggregate to a structure that resembled a spheroid. Within the same time frame, C-FR1 cells formed an aggregate displaying a spheroid-like structure in its center. C-FR2 and C-FR3 were completely unable to form spheroid-like structures. The higher levels of P-STAT3 and integrin β1 in the C-FR cells are the likely reasons for the higher defect in spheroid formation as displayed by the C-FR sublines.

Collectively, these data suggest that the presence of CAF-CM during the establishment of FR sublines resulted in permanently high expression of integrin β1, P-STAT3 and Bcl-3. In spite of the high levels, at which these three proteins involved in drug resistance are expressed, CAF-CM-treated FR cells do not show higher fulvestrant resistance than FR cells that were exposed to control CM. Nor did CAF-CM treatment fostered cell growth or migration of FR cells. In the contrary, C-FR3 cells required the presence of CAF-CM to migrate as fast as M-FR cells in the absence of CM, again suggesting addiction to CAF-CM. There may, however, be an effect of long-term CAF-CM treatment on spheroid formation as C-FR cells were less able to form spheroids than M-FR cells.

### C-FR cells became addicted to integrin β1

**Figure 6:**
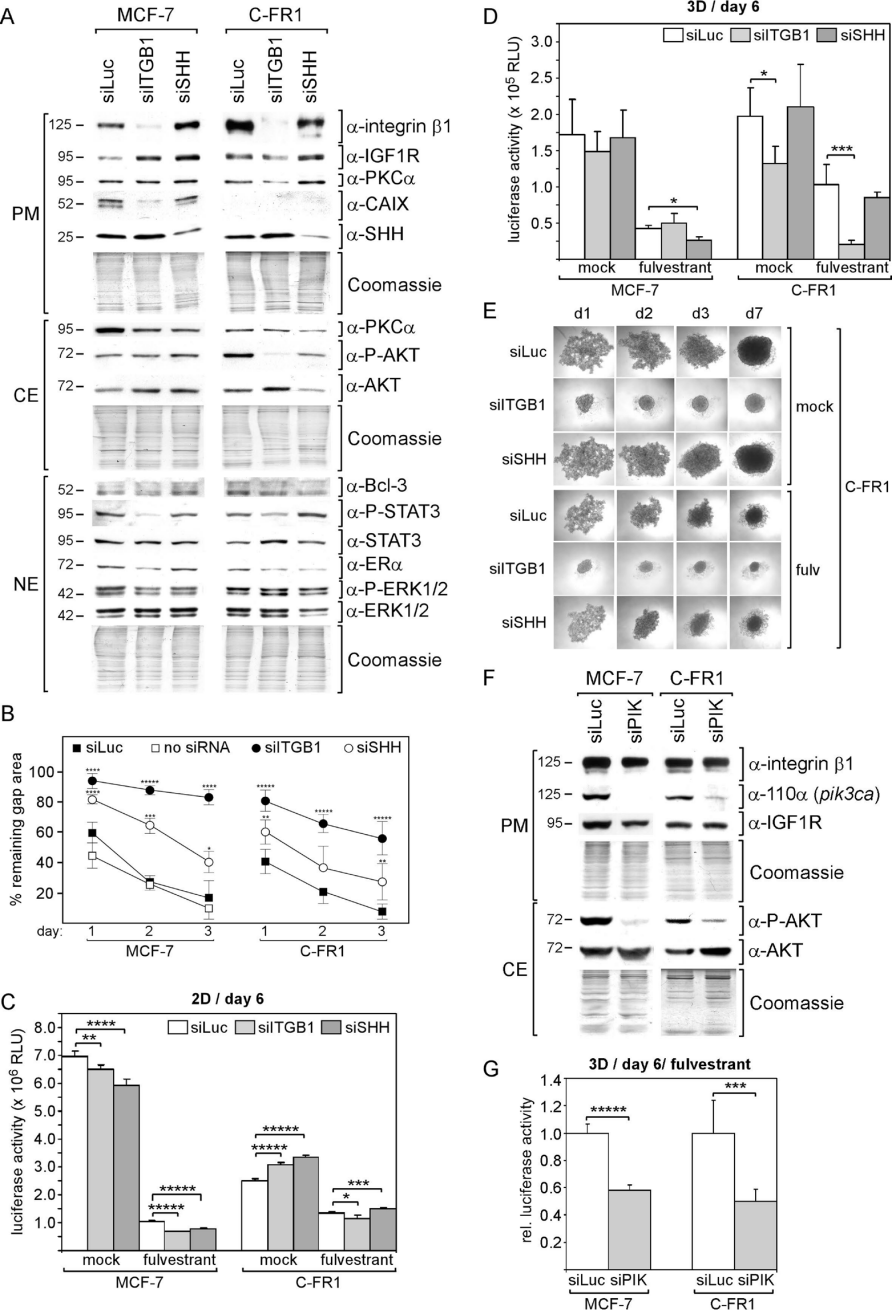
Cells that developed fulvestrant resistance in the presence of CAF-CM require integrin β1 to withstand fulvestrant in 3D cultures (**A, F**) Western blot analysis of plasma membrane (PM), cytosolic (CE) and nuclear (NE) protein extracts from MCF-7 and C-FR1 cells transfected with the siRNA as indicated. Proteins were isolated five days after transfection. Blots were probed with the antibodies as indicated. Total protein applied was checked by Commassie staining. (**B**) Wound healing assay. Cells were transfected with siRNA or no siRNA and incubated for three days before wound had been set. Gap closure was monitored for the following three days. (**C, D, G**) Growth assays of cells transfected with the siRNA as indicated in the absence or presence of 1 μM fulvestrant in 2D adherent (C) or 3D suspension cultures (D, G). After transfection cells were incubated for three days in 2D culture before cells were seeded for a 2D or 3D growth assay. Cell mass was then measured after six days of incubation. (**E**) Spheroid formation of C-FR1 cells in 3D suspension cultures following transfection with the siRNA as indicated. After transfection cells were first kept in 2D cultures for three days, before they were exposed to 3D cultures conditions. Spheroid formation was monitored for seven days.

To explore the importance of the high level of integrin β1 for the cellular activities of C-FR cells, we performed RNA interference experiments. An integrin β1-specific siRNA (siITGB1) was used along with a control siRNA targeting firefly luciferase (siLuc) and an SHH-specific siRNA (siSHH). For this study, we chose the subline C-FR1, which shows the highest expression of integrin β1 (Figure 5A). For comparison, the experiments were also performed with the MCF-7 parental cell line. Western blot analyses demonstrated that siITGB1 completely erased integrin β1 protein expression in both cell lines (Figure 6A). Along with it, the P-STAT3 levels were reduced, an observation which is in agreement with a previous study showing involvement of integrin β1 in the regulation of STAT3 phosphorylation in myeloma cells [21]. In C-FR1 cells, siITGB1 also blocked AKT phosphorylation, which was not observed with MCF-7 cells, though both cell lines express the same level of P-AKT (Figure 5A). In MCF-7 cells, siITGB1 strongly reduced the level of CAIX, a protein not expressed in the FR cells.

To assess the effect of siITGB1 on biological activities of the cells, growth in 2D and 3D cultures, migration and spheroid formation abilities were assessed. In wound healing assays, both siITGB1 and siSHH strongly delayed gap closure of C-FR1 and MCF-7 cells, whereby siITGB1 was more effective than siSHH (Figure 6B). In terms of growth, the effect of siITGB1 strongly depended on whether cells were grown in 2D or 3D cultures. In 2D cultures, sITGB1 and also siSHH had weak to moderate effects on C-FR1 and MCF-7 cell growth (Figure 6C). However, in 3D cultures, siITGB1 only affected growth of C-FR1 cells (Figure 6D). SiITGB1 significantly decreased growth of C-FR1 cells in the presence and absence of fulvestrant, whereby this effect was much stronger in the presence of fulvestrant. Knock-down of integrin β1 also dramatically changed spheroid formation pattern of C-FR1 cells and allowed these cells to aggregate to well-shaped spheroids within two days in the presence and absence of fulvestrant (Figure 6E). We wondered if the strong siITGB1 effect on C-FR1 cells in 3D cultures is linked to the siITGB1-induced loss of P-AKT in these cells. To test this, we downregulated p110α, the catalytic subunit of PI3K encoded by the gene *pik3ca*, by a PIK3CA-specific siRNA (siPIK), which resulted in a substantial reduction in P-AKT levels in both C-FR1 and MCF-7 cells (Figure 6F). Along with these changes, siPIK caused a significant retardation in 3D cell growth of both cell lines, when studied in the presence of fulvestrant (Figure 6G).

These data suggest that the permanently high expression of integrin β1 in C-FR cells is required to allow PI3K/AKT pathway-dependent growth activity in 3D cultures whereas MCF-7 parental cells do not rely on integrin β1 for this activity. This suggest that long-term treatment with CAF-CM during development of fulvestrant resistance makes cells addictive to integrin β1 to maintain certain cellular activities to the same extent as before CAF-CM treatment.

## DISCUSSION

Previous data reported by us and others have shown that carcinoma-associated fibroblasts protect breast cancer cells against anti-estrogens [4, 6, 22]. In all cases, treatment with CAFs was limited to 1 to 3 days. In our present study, we extended the incubation time to several weeks. This required us to do the analysis with MCF-7 sublines established from fulvestrant-tolerating dormant and fulvestrant-resistant cells. Unexpectedly, long-term treatment of these cells with CAF-CM did not improve protection against fulvestrant. In the absence of CAF-CM, they were even much more sensitive to fulvestrant than cells that were treated with control CM. Hence, in contrast to short-term exposure, long-term exposure to CAF-CM does not help breast cancer cells to better cope with fulvestrant. The protecting effect of short-term CAF-CM treatment relies on the protein Bcl-3 [4]. Long-term treatment with CAF-CM did not increase Bcl-3 expression in fulvestrant-resistant cells above the level of MCF-7 cells, but kept it higher than that in control CM-treated counterparts (Figure 5A). Only in AnD5/C-D cells increased Bcl-3 levels were found but without apparent effect on fulvestrant tolerance. In long-term fulvestrant resistance, Bcl-3 does not seem to be essential, as control CM-treated cells (M-FR1 and M-FR2) developed fulvestrant resistance at barely detectable or low levels of Bcl-3.

Long-term treatment with CAF-CM also failed to induce better performance in two other cellular activities, namely growth in the absence of fulvestrant and migration. The missing effect on migration was again unexpected, since short-term treatment of CAF-CM substantially stimulate migration in most cases. Migration seems to be heavily dependent on integrin β1 (Figure 6B). However, C-FR cells, which express high levels of integrin β1, did not perform better in the migration assays than M-FR or MCF-7 cells, which express much less of this protein (Figures 5A, 5D). For example, M-FR2 cells migrate as fast as C-FR-1 cells, though M-FR2 show barely detectable integrin β1 levels and C-FR-1 express the highest level of integrin β1 of all FR sublines.This suggests that a certain level of integrin β1 is required for migration, above that no further increase in migration is achieved. An effect that may be ascribed to long-term CAF-CM treatment on cellular activity is on cell aggregation in 3D cultures. There was a tendency that fulvestrant-resistant cells that were exposed to CAF-CM showed a lower activity in cellular aggregation than FR cells exposed to control CM (Figure 5E). The defect in spheroid formation ability of C-FR1 cells could be shown to be caused by their high expression of integrin β1 (Figure 6B).

Although long-term treatment with CAF-CM had no obvious effects on migration, cell growth and fulvestrant resistance, it had dramatic, long-lasting effects on protein expression and signaling pathway activities. In all cases, long-term treatment with CAF-CM led to a permanent increase in the expression of integrin β1. In FR cells, also Bcl-3 and P-STAT3 levels were permanently increased upon CAF-CM pre-treatment and, in C-D cells, it was IGF1R which was upregulated along with integrin β1. All these proteins have the potential to stimulate migration, proliferation and/or drug resistance [4–6, 23–31] and we confirmed here that integrin β1 is a key regulator of MCF-7 cell migration (Figure 6B). Hence, it is surprising that the high expression of these proteins in the CAF-CM-treated cells did not endow these cells with better performance in migration, growth and fulvestrant resistance. A likely explanation of this apparent contradiction is that the CAF-CM-treated cells just got addicted to the high levels of these proteins without gaining advantage, at least not in any of the cellular activities we have tested. This notion is supported by the finding that growth of C-FR1 cells in 3D cultures is dependent upon integrin β1, whereas it is not in MCF-7 parental cells, though both cell lines show similar growth activities under these conditions (Figure 6D). The dependency of C-FR1 cell growth in 3D culture on integrin β1 is even more pronounced in the presence of fulvestrant, whereas again this protein is dispensable for MCF-7 parental cells to grow under these conditions. We present evidence that MCF-7 and C-FR1 cells depend on the PI3K/AKT pathway for growth in 3D cultures in the presence of fulvestrant and that, only in C-FR1 cells, the PI3K/AKT pathway is dependent on integrin β1. This suggests that, in C-FR cells, CAF-CM-induced integrin β1 became an integral part of the PI3K/AKT pathway regulation, which is not the case in parental MCF-7 cells.

While addiction is a plausible explanation for the data we have obtained, we cannot rule out the possibility that the overexpressed factors in the CAF-CM-treated cells lead to better performances in cellular activities we have not tested, such as metastatic and immunosuppressive activities. For example, integrin β1 has been shown to be involved in the switch between dormancy and active proliferation at metastatic sites [32], thereby promoting the formation of macrometastatic lesions. Integrin β1 also enhances metastatic activity of breast cancer cells by facilitating extravasation [33]. P-STAT3 expressed by breast cancer cells was found to repress immunosurveillance and thereby promote tumor progression [34].

We found that long-term treatment of breast cancer cells resulted in permanently increased expression of certain proteins. These high levels were maintained even in the absence of CAF-CM showing that the presence of CAF-CM was no longer needed. This suggests that CAF-CM permanently changed signaling pathway activities and/or induced epigenetic changes. The high P-STAT3 levels found along with the high integrin β1 levels in the C-FR sublines and the finding that integrin β1 regulates STAT3 phosphorylation may explain how long-term CAF-CM treatment has stimulated the JAK/STAT3 signalling pathway activity. Long-term effects of CAFs on protein expression in epithelial and carcinoma cells has also been reported by other groups. Lin *et al.* showed that long-term co-cultures of breast CAFs with the MCF10A cells resulted in silencing of the tumor suppressor gene cytostatin M due to increased activation of AKT [35]. This effect required direct cell-cell contact. In another study, co-cultures of gastric cancer cells with gastric CAFs gave rise to increased methylation of miR-200b, leading to lower expression of this EMT (epithelial-to mesenchymal transition)-regulating microRNA and poorer prognosis [36]. Recently, Pistore *et al.* demonstrated that CAF-CM can induce changes in the DNA methylation pattern in prostate cancer leading to EMT [37]. Gene silencing can also occur in CAFs after co-culture with carcinoma cells. Xiao *et al.* reported that pancreatic carcinoma cells are able to induce promoter methylation of the SOCS1 gene in CAFs [38]. Also, breast cancer cells have been shown to force normal tissue-associated fibroblasts to permanently produce the invasion-promoting protease ADAMTS1 (a disintegrin and metalloproteinase with thrombospondin motifs 1). This was accompanied by decreased histone 3 K27 methylation at the ADAMTS1 promoter, a change that persisted even after removal of the breast cancer cells [39]. These examples support the notion that environmental conditions can permanently change gene expression based on epigenetic changes. Hence, the changes in protein expression we have observed after long-term treatment with CAF-CM could have been caused by epigenetic changes as well. On the other hand, it cannot be ruled out that exposure to CAF-CM initialized a selection process, in which those cells grew out which could cope best with the presence of the many growth factors and cytokines present in CAF-CM. Such a selection process was shown for triple-negative MDA-MB-231 breast cancer cells that were exposed to CAFs [7]. Under the influence of CAF-secreted IGF1 and SDF-1 (stromal-derived factor-1) a subpopulation of cancer cells that expressed the IGF1 receptor IGF1R and the SDF-1 receptor CXCR4 outgrew other cancer cell subpopulations. This was shown to have consequences for metastasis, as IGF1R/CXCR4-expressing breast cancer cells have a higher potential to metastasize to bone.

Our data also show that there are at least two morphologically distinct subpopulations within the MCF-7 cell line. The majority of MCF-7 cells is made up by a highly motile cell-type, which we called AnD5 cells, whereas the less motile AnD3 cell-type is much less abundant in the MCF-7 cell population. MCF-7 cell line heterogeneity has also been reported by others [40, 41]. With some breast cancer cell lines, heterogeneity has been demonstrated to be caused by interconversion of cancer cells between different states [42]. However, there is no evidence that AnD3 cells interconvert to AnD5 cells and *vice versa* (data not shown) suggesting that the AnD3 and AnD5 populations are distinct and stable subpopulations of the MCF-7 cell line. In terms of their reactivity to short-term exposure to CAF-CM, AnD3 and AnD5 cells share key responses, such as upregulation in Bcl-3 expression and increased growth in fulvestrant-containing medium. Also, sublines established from CAF-CM-treated AnD3 and AnD5 dormant cells show both permanently elevated expression of integrin β1 and IGF1R expression and higher sensitivity to fulvestrant compared to their counterparts exposed to control CM. However, particularly when given short-term, there are also differences in the reactions of AnD3 and AnD5 cells to CAF-CM, including different patterns in protein expression changes and a different extent by which migration is stimulated by CAF-CM. Hence, though AnD3 and AnD5 cells are different in many features, including morphology, migration, growth activity and expression of a number of tumor-relevant proteins, they share major responses to CAF-CM. In contrast, a comparison between the MCF-7 cell line with another ERα-positive breast cancer cell line, T47D, revealed almost no similarities in the responses to CAF-CM other than an increase in STAT3 phosphorylation [4]. This suggests that, in terms of responses to stromal cells, intertumoral differences are more pronounced than intratumoral differences.

In conclusion, these data show that breast cancer cells challenged by fulvestrant in an environment containing CAF-secreted factors permanently increase the expression of certain tumor-relevant proteins, particularly integrin β1. The data further suggest that the cells become addictive to these proteins without deriving a benefit from them in terms of fulvestrant tolerance, growth and migration. Nevertheless, given the potential importance of these proteins in tumor progression, it might be beneficial for the cells to have them at some point in the course of tumor development. The data also suggest that, though intratumoral heterogeneity increases the complexity of responses of breast cancer cells to CAF-CM, major reactions to CAF-CM are shared by the different subpopulations.

## MATERIALS AND METHODS

### Cell lines/sublines

MCF-7 and all sublines were authenticated by SNP analysis (Genolytic, Leipzig, Germany). Immortalized 19TT breast carcinoma-associated fibroblasts are described previously [4]. All cells were maintained in RPMI medium supplemented with 10% fetal calf serum (FCS, Pan Biotech, Germany) in the absence of antibiotics. For all cell lines, the same batch of FCS was used. CAF-CM was obtained from 19TT CAFs, MCF-CM from MCF-7 parental cells. For the preparation of CM, CAFs or MCF-7 cells were trypsinized and grown for three days, before the medium/FCS was replaced by fresh medium/FCS, which was removed after three more days and centrifuged at 3000 rpm/min in a Multifuge 3 (Heraeus) for 15 min to pellet floating cells. The supernatant was used as CM after it was filtered through a 0.45 µm Minisart NML Syringe Filter (Sartorius). CM was then added to fresh medium/FCS at the following ratios: 5 + 95 (5% CM), 10 + 90 (10% CM) or 20 + 80 (20% CM). MCF-7 sublines AnD3 and AnD5 were established by seeding MCF-7 cells at low density (5 × 10^4^ cells per Ø 10 cm cell culture dish) and picking morphologically distinct colonies for further propagation (Supplementary Figure 1). Fulvestrant-resistant (FR) sublines were generated by seeding MCF-7 cells at low density and exposing them to 1 µM fulvestrant (LKT Laboratories) in the presence of either 20% CAF-CM or 20% MCF-CM while replacing the medium weekly. After four weeks, large proliferating colonies could be observed. From two individual colonies grown up in the presence of MCF-CM, sublines called M-FR1 and M-FR2 could be established, from three colonies grown up in the presence of CAF-CM, sublines called C-FR1, C-FR2 and C-FR3 were generated. For further propagation, M-FR and C-FR cells were grown in fulvestrant-containing medium supplemented with MCF-CM or CAF-CM, respectively. To obtain sublines from fulvestrant-treated AnD3 and AnD5 cells in the presence of MCF-CM or CAF-CM, cells were treated the same way as described above for MCF-7 cells (Supplementary Figure 1). However, in contrast to MCF-7 parental cells, AnD3 and AnD5 cells failed to generate proliferating colonies in the presence of fulvestrant even after six weeks of incubation. Though not proliferating, many cells were found to stay attached to the substratum suggesting that they were still alive and entered dormancy to better tolerate fulvestrant [11]. After withdrawal of fulvestrant, some colonies formed and were picked for further propagation. Sublines generated from these individual colonies exposed to CAF-CM or MCF-CM were called C-D(ormant) or M-D to distinguish them from the C-FR and M-FR sublines, respectively. Three M-D sublines (AnD3/M-D1, /M-D2 and /M-D3) and two C-D sublines (AnD3/C-D1 and C-D2) could be established from AnD3 cells. AnD5 cells gave rise to two M-D sublines (AnD5/M-D1 and /M-D2) and one C-D subline (AnD5/C-D). M-D or C-D cells were propagated in MCF-CM- or CAF-CM-containing medium, respectively, in the absence of fulvestrant. For spheroid formation in 3D suspension cultures, cells were incubated on a layer of 2% Seakem GTG agarose (dissolved in PBS) in 96-well plates at a density of 5 × 10^3^ cells/well for up to seven days. To measure the spheroid size, a picture was taken by an AxioCam MRc 5 camera (Zeiss, Jena, Germany) and the area displayed on this picture measured by AxioVision R 4.5 (Zeiss, Jena, Germany) software as described previously [43].

### RNA interference

Small interference (si)RNAs were purchased from Eurofins MWG. Transfection was performed by electroporation as described [44]. Briefly, cells were trypsinized, washed once in RPMI medium, electroporated by using a Bio-Rad GenePulserX-Cell at 250V and 800µF and kept on ice for 30 min. Cells were then transferred to a 10 cm culture dish and incubated for three days to allow the siRNA to downregulate the expression of its target. The effect of the siRNA was confirmed by Western blot analysis. For analyzing siRNA effects on cells in spheroids, cells were transfected with the siRNA, incubated for three days in adherent cultures, trypsinized and then grown in 3D suspension cultures. This procedure allows the siRNAs to be active for at least another four days after transferral of cells to the suspension culture [43]. The following siRNAs were used: integrin β1-specific siRNA siITGB1 (5′-AAG ACU GUG AUG CCU UAC A -3′), SHH-specific siRNA siSHH (5′-CCA GAC UGA GUU AUA AUA A -3′), p110α (*pik3ca*)-specific siRNA siPIK (5′-GUA CAG GAC UUC CGA AGA A-3′) and control, firefly luciferase-siRNA, siLuc (5′-CUU ACG CUG AGU ACU UCG A-3′).

### Growth assays

Cell growth activity was determined by an ATP-based assay (Vialight Plus Kit, Lonza). Cells were seeded at a density of 1 × 10^4^ per well of a 24-well plate and incubated in the presence of fulvestrant and/or CAF-CM or left untreated for six days. After removal of the growth medium, a mixture of 100 µl PBS and 50 µl lysis buffer was added to the cells and the suspension rotated for 10 min. The lysate was mixed with 50 µl luciferase stock solution and luciferase activity measured in a Sirius luminometer (Berthold). Growth assays at low cell density were performed as follows. After seeding cells at 3 × 10^4^ cells per 10 cm dish, they were either incubated with CAF-CM and/or 1 µM fulvestrant or left untreated for six days. Sizes of individual colonies were then measuring by using an AxioCAM MRc5 camera and the AxioVision R 4.5 imaging software (Zeiss, Jena). For each condition, the average size of fifty randomly chosen colonies was determined.

### Proliferation assay

Proliferation was determined by incorporation of BrdU (5-bromo-2´-deoxyuridine) into DNA as described [44]. Briefly, 2500 cells per well were seeded into 96-well plates and incubated with BrdU for 24 h, after which BrdU incorporation was measured by an anti-BrdU ELISA (Roche).

### Quantitative RT-PCR

RNA isolation, cDNA synthesis and quantitative (Q) PCR were carried out as described [4]. Briefly, for cDNA synthesis, Superscript II (Invitrogen) was used. Q-PCR was run in a BioRAD iCycler by using ABsolute QPCR SYBR Green Fluorescein Mix (Thermo Fisher Scientific Biosciences). Results were analyzed by iQ5 Optical System software version 2.1. Relative RNA levels of genes were calculated by the comparative *Ct* (2^-∆∆*Ct*^) method by using GAPDH and HPRT as reference genes for normalization [44, 45]. The primers used for detection of vimentin and E-cadherin are as follows: vimentin (forward 5′^→^3′: GCAGGAGGCAGAAGAATGGTA, reverse 5′^→^3′: CAGCCTCAGAGAGGTCAGCAA), E-cadherin (forward 5′^→^3′: TTGACTTGAGCCAGCTGCAC, reverse 5′^→^3′: CGTTACGAGTCACTTCAGGCC).

### Migration assays

Migration activity was studied by wound healing and/or Boyden chamber assays as described [46]. Briefly, for the wound healing assay, a gap was introduced into a monolayer of breast cancer cells. Gap closure in the presence of absence of 20% CAF-CM was monitored for up to three days. For quantitation, the gap area as visible at 100-fold magnification was measured by using Zeiss Axio Vision R 4.5 software. For each condition, the average gap area of at least three independent wounds were determined. In the Boyden chamber assay, cells were seeded on the upper compartment of a Boyden chamber and allowed to migrate through the 8-µm-pore filter. After three days, cells that remained on the upper side of the filter were wiped off and cells that migrated to the lower side of the filter were fixed, stained with H&E and counted under a microscope (20 fields per filter).

### Western blot analysis

Protein extractions and Western blot analysis were carried out as described [4]. Briefly, cells were scraped off the plate, centrifuged and resuspended in 400 μl buffer A (10 mM HEPES (pH 7.9), 10 mM KCl, 0.1 mM EDTA, 0.1 mM EGTA). After having passed the suspension through a 20-gauge needle five-times, the lysate was fractionated by stepwise centrifugation at 3000, 6500 and 13000 rpm in a microfuge to obtain cytosolic, nuclear and plasma membrane protein extracts. For nuclear or plasma membrane protein extraction, the pellet was extracted in buffer C (20 mM HEPES (pH 7.9), 400 mM NaCl, 1 mM EDTA, 1 mM EGTA, 1 mM DTT) or buffer D (5 mM HEPES (pH 7.9), 0.5 mM K-EDTA (pH 7.2), 1 mM DTT), respectively. Protein extracts were separated on a 10% SDS-polyacrylamide gel and transferred to a PVDF membrane (Millipore). After blocking the membrane in 2% skim milk (Applichem) dissolved in washing buffer (10 mM Tris/HCl (pH 7.5), 100 mM NaCl, 1 mM EDTA), it was sequentially incubated with the primary antibody and the secondary antibody in washing buffer containing 0.2% skim milk. Peroxidase activity was visualized by chemoluminescence using ECLPlus and Hyperfilm ECL (GE Healthcare). For Western blot analysis, the following antibodies were used (working dilutions are given in brackets). Rabbit polyclonal antibodies: anti-P(S473)-AKT (1:2000, D9E, Cell Signaling), anti-Bcl-3 (1:1000, C-14, Santa Cruz), anti-P(Thr202, Tyr204)-ERK1/2 and anti-ERK1/2 (both 1:2000, Cell Signaling), anti-ERα (1:2000, Santa Cruz, HC-20), anti-IGF1Rβ (1:2000, Cell Signaling), anti-PKCα (1:2000, Santa Cruz, C-20), anti-SHH (1:1000, Santa Cruz, H-160), anti-P(Tyr705)-STAT3 (1:1000, D3A7, Cell Signaling) and anti-STAT3 (1:1000, 79D7, Cell Signaling); rabbit monoclonal antibodies: anti-integrin β1 (1:2000, EPR1040Y, Abcam); mouse monoclonal antibodies: anti-(pan)AKT (1:1000, 40D4, Cell Signaling) Anti-CAIX was kindly provided by S. Pastorekova. Secondary antibody conjugates (anti-rabbit/anti-mouse horse radish peroxidase, 1:2000) were purchased from Cell Signaling. Protein loading was checked by either staining proteins by Coomassie Blue (Blue G, Serva) or by Fast Green. Antibodies against housekeeping proteins were not used for this purpose, since they are not reliable markers for protein loading [47, 48].

### Statistical analyses

Data obtained from colony growth assays were analyzed by Mann-Whitney-U-test. All other statistical analyses were done by using the student *t*-test. A *p*-value of *p* < 0.05 was considered to be statistically significant. For all graphs: ^*^p < 0.05, ^**^p < 0.01, ^***^p < 0.005, ^****^p < 0.0005, ^*****^p < 0.0001.

## SUPPLEMENTARY MATERIALS FIGURES


