# Supplementary material for: Long-term exposure to carcinoma-associated fibroblasts makes breast cancer cells addictive to integrin β1
Source: Oncotarget. 2018 Apr 24;9(31):22079–94. doi: 10.18632/oncotarget.25183 (PMC5955132; doi:10.18632/oncotarget.25183)
Supplement: Supplementary file 1 [file oncotarget-09-22079-s001.pdf]

Long-term exposure to carcinoma-associated fibroblasts makes breast cancer cells addictive to integrin β1

SUPPLEMENTARY MATERIALS

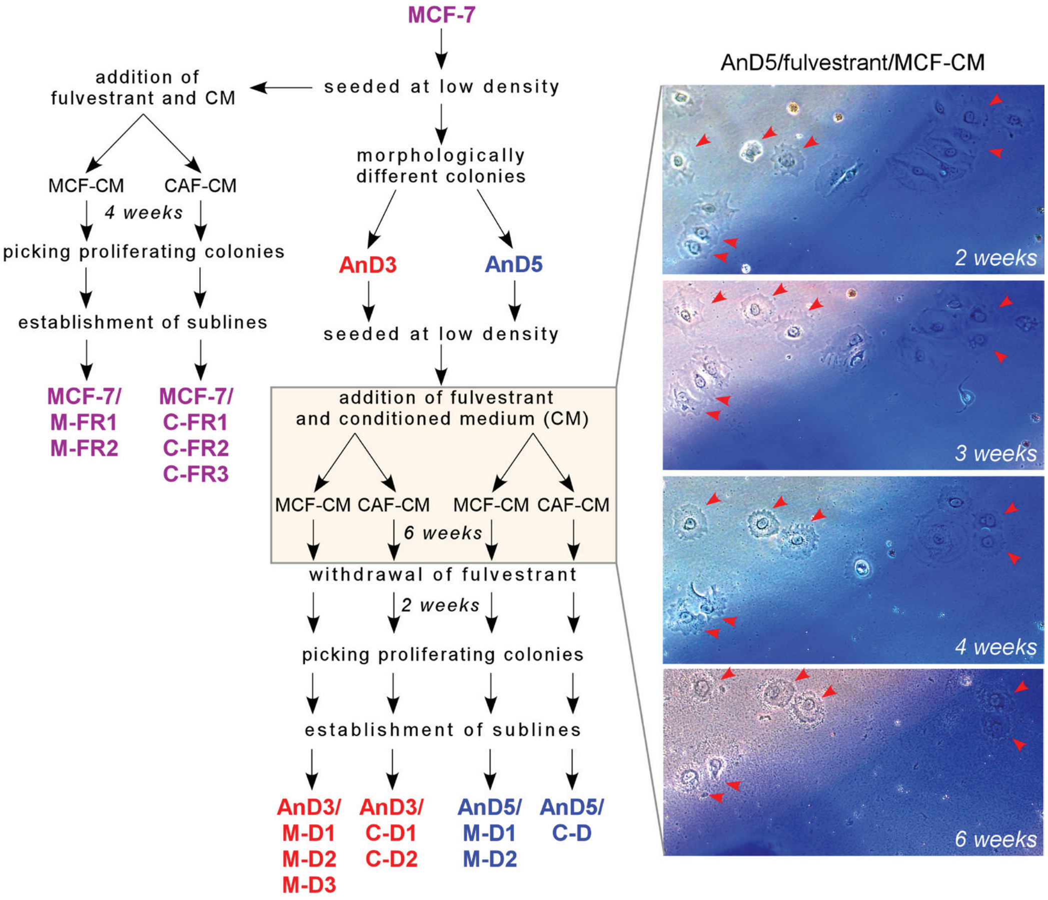

Supplementary Figure 1: A schematic depicting the generation of the different MCF-7 sublines used in this study. The right panel shows phase contrast images of the same AnD5 cells after 2, 3, 4 and 6 weeks of exposure to 1 µM fulvestrant and MCF-CM. Most cells died and disintegrated. Arrow heads indicate those cells that were still visible after the six-week-treatment. C-D = CAF-CM treated Dormant cells, M-D = MCF-CM treated Dormant cells, C-FR = CAF-CM treated Fulvestrant-Resistant cells, M-FR = MCF-CM treated Fulvestrant-Resistant cells.

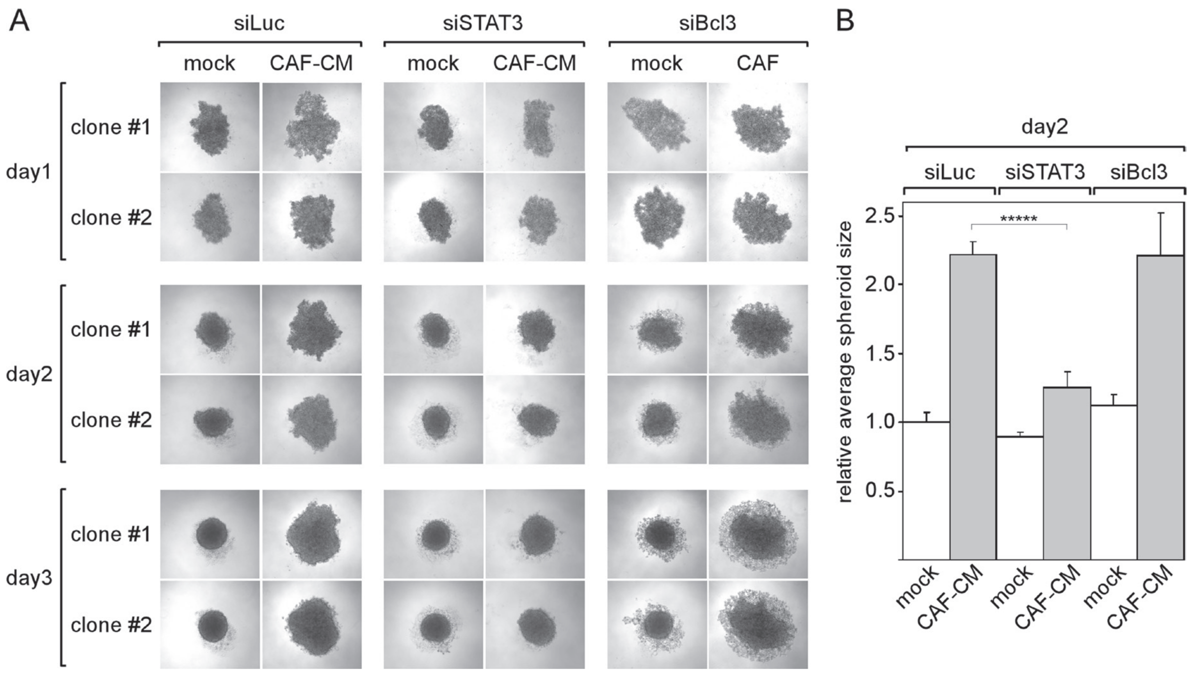

Supplementary Figure 2: STAT3 is responsible for the CAF-CM-induced defect in proper spheroid formation. MCF-7 cells were transfected with an STAT3-specific siRNA (siSTAT3), a Bcl-3 targeting siRNA (siBcl3) or a control siRNA (siLuc), incubated in 2D adhesion cultures for three days, trypsinized and transferred to 3D suspension cultures, where cell aggregation was monitored for three days (**A**). Spheroid size was determined by using the AxioVision R 4.5 software (Zeiss, Jena) at day 2 (**B**).The siRNAs are decribed elsewhere (Leyh et al. Oncotarget 2015, 6: 39307)
